# Experimental Autoimmune Encephalomyelitis Influences GH-Axis in Female Rats

**DOI:** 10.3390/ijms25115837

**Published:** 2024-05-27

**Authors:** Anica Zivkovic, Svetlana Trifunovic, Danijela Savic, Katarina Milosevic, Irena Lavrnja

**Affiliations:** 1 Department of Neurobiology, Institute for Biological Research “Sinisa Stankovic”—National Institute of Republic of Serbia, University of Belgrade, 11108 Belgrade, Serbia; anica.zivkovic@ibiss.bg.ac.rs (A.Z.); danisto@ibiss.bg.ac.rs (D.S.); katarina.tesovic@ibiss.bg.ac.rs (K.M.); 2 Department of Cytology, Institute for Biological Research “Sinisa Stankovic”—National Institute of Republic of Serbia, University of Belgrade, 11108 Belgrade, Serbia; lanat@ibiss.bg.ac.rs

**Keywords:** EAE, growth hormone, IGF-1, pituitary

## Abstract

Inflammation, demyelination, and axonal damage to the central nervous system (CNS) are the hallmarks of multiple sclerosis (MS) and its representative animal model, experimental autoimmune encephalomyelitis (EAE). There is scientific evidence for the involvement of growth hormone (GH) in autoimmune regulation. Previous data on the relationship between the GH/insulin like growth factor-1 (IGF-1) axis and MS/EAE are inconclusive; therefore, the aim of our study was to investigate the changes in the GH axis during acute monophasic EAE. The results show that the gene expression of *Ghrh* and *Sst* in the hypothalamus does not change, except for *Npy* and *Agrp*, while at the pituitary level the *Gh*, *Ghrhr* and *Ghr* genes are upregulated. Interestingly, the cell volume of somatotropic cells in the pituitary gland remains unchanged at the peak of the disease. We found elevated serum GH levels in association with low IGF-1 concentration and downregulated *Ghr* and *Igf1r* expression in the liver, indicating a condition resembling GH resistance. This is likely due to inadequate nutrient intake at the peak of the disease when inflammation in the CNS is greatest. Considering that GH secretion is finely regulated by numerous central and peripheral signals, the involvement of the GH/IGF-1 axis in MS/EAE should be thoroughly investigated for possible future therapeutic strategies, especially with a view to improving EAE disease.

## 1. Introduction

Multiple sclerosis (MS) is a debilitating, chronic inflammatory disease of the central nervous system (CNS) that causes severe neurological disability in young adults. The course of the disease is variable and unpredictable, and symptoms vary from person to person [1]. Because of the heterogeneity of the symptoms and clinical course of MS, there is no single animal model that could parallel the full spectrum of the disease [2]. A single cause of MS is not known, but immunological, genetic/epigenetic, and environmental factors have been shown to contribute to the development of the disease. Therefore, various animal models have been used in experiments to mimic certain aspects of MS pathology, particularly the acute inflammatory stage, as experimental autoimmune encephalomyelitis (EAE) [3,4]. EAE is triggered by an autoimmune attack against myelin mediated by autoreactive CD4+ T cells through injection of various myelin peptides emulsified in an adjuvant.

It has already been shown that alterations in the complex system of neuroendocrine pathways and feedback loops may accompany the progression of MS and EAE [5,6]. Numerous studies suggest that the hypothalamic-pituitary-adrenal (HPA) axis plays a role in both disease susceptibility and recovery from an MS relapse [7,8], primarily through alterations in cortisol concentrations [9]. In addition, prolactine and sex hormones, such as estrogens, progesterone, and testosterone, are thought to be involved in regulating the course of MS [10]. However, complex interactions between the growth hormone (GH) and insulin-like growth factor-1 (IGF-1) axis on immune function in MS have been confirmed but not fully explained [11]. The GH axis refers to the hormonal pathways that regulate the synthesis, release, and action of growth hormone in the body, involving the hypothalamus, pituitary gland, and target tissues. The secretion of pituitary GH to the bloodstream is positively regulated by hypothalamic GH-releasing hormone (GHRH) and negatively by hypothalamic somatostatin (SST). GH exerts its effects through interaction with the GH receptor (GHR), a member of the class I cytokine receptor family that regulates GH availability [12]. Once GH is released into the bloodstream, it exerts its effects on various tissues throughout the body, particularly the liver, where it stimulates the production of IGF-1. Besides their primary role in regulating various physiological processes [13], GH and IGF-1 play essential roles in the development, differentiation, and function of the immune system [14,15,16]. GH and IGF-1 receptors are present on immune cells, indicating their direct role in immune regulation [14]. It is known that the relationships between the immune system and the GH/IGF-1 axis are reciprocal, and both GH and IGF-1, the main mediators of its action, could modulate the inflammatory response and the activity of systemic inflammation [4,11,12,13]. The available data suggest that the GH/IGF-1 axis has both proinflammatory and anti-inflammatory effects [4,11,14,15,16,17]. While the specific role of GH/IGF-1 in autoimmune disease development is not fully understood, there is some evidence to suggest that GH may play a role in modulating immune function, which could potentially influence autoimmune disease development [17,18,19,20].

Previously, Ikushima et al. demonstrated that GHRH is required for the development of experimental autoimmune encephalomyelitis [17]. Later, GH and not GHRH was shown to play a crucial role in the development of EAE [18]. In MS patients, decreased levels of GH were found in the cerebrospinal fluid (CSF), suggesting a disturbance of the GH/GHRH regulatory circuit [21]. Later, it was shown that there were also no significant differences in CSF GH levels in men and women with MS [22], while Poljakovic et al. found lower CSF GH levels in patients with MS [23]. In addition, it has been proposed that low IGF-1 levels may be associated with susceptibility to MS [24].

Thus, the reported data on endocrine alterations of the GH/IGF-1 axis in MS/EAE are inconclusive or contradictory [11,17,18,23,25]. To our knowledge, no previous study has examined the association of the hypothalamic-pituitary-GH axis, particularly GH/IGF-1 and its accompanying receptors, during acute monophasic EAE. In this study, we highlight the expression of genes and proteins involved in the GH axis during acute inflammatory EAE disease.

## 2. Results

### 2.1. Exacerbation of EAE Severity Coincides with Body Weight Loss

**Figure 1:**
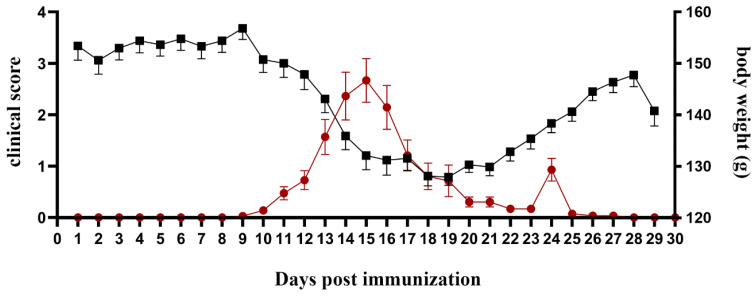
Acute EAE—clinical course and weight loss. Acute EAE is induced via subcutaneous immunization with spinal cord homogenate in complete Freund’s adjuvant. Clinical course of disease is scored daily on a scale of 0 to 5 (*Y*-axis left, red-round shape). Body weight was presented at *Y*-axis (right, black-square shape). Clinical signs of disease are usually first observed between days 10 and 15, followed by monophasic disease characterized by severe walking disability, with substantial weight loss followed by spontaneous recovery.

Using the standard EAE scoring system, DA rats showed a gradual increase in clinical EAE scores beginning on days 10–13 (disease onset, Eo) with complete loss of tail tonicity, followed by hind limb paralysis (peak of disease, Ep) on days 12–17 (Figure 1), and complete recovery (end of disease, Ee). The mean day of disease onset was 11 dpi (11.56 ± 0.97), and the other parameters of disease severity are shown in Table 1. During disease development, a reduction in weight was observed (Figure 1).

### 2.2. Hypothalamic Expression of Genes Associated with GH Axis

**Figure 2:**
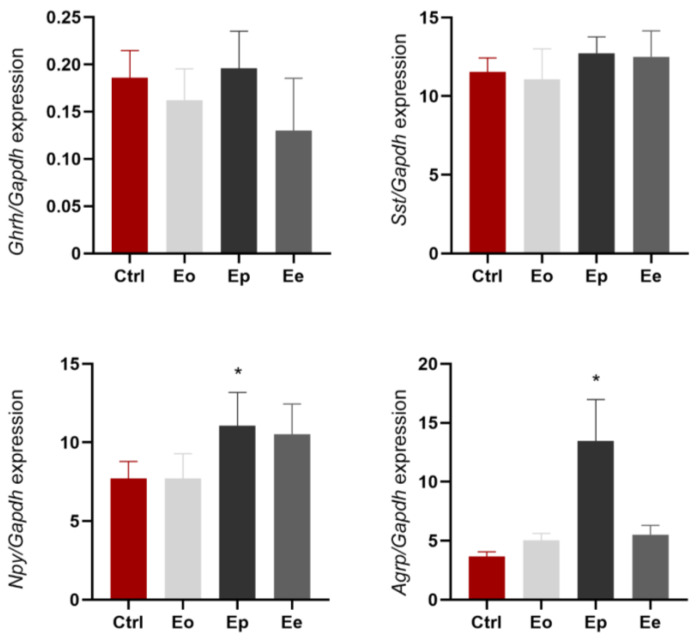
EAE affects relative gene expression of *Ghrh*, *Sst*, *Npy*, and *Agrp* in the hypothalamus. Levels of target genes are expressed relative to the expression of the *Gapdh* gene. Expression profiles were analyzed via RT-qPCR from RNA isolated from animals at disease onset (Eo, light gray bars), disease peak (Ep, dark gray bars), disease end (Ee, gray bars), and the control animals (Ctrl, red bars). The data shown are mean ± SEM (n ≥ 5 animals/group), derived from a representative experiment. There are no significant changes in the *Ghrh* and *Sst* expression. *Npy* and *Agrp* expression increase at the peak of disease. The results were analyzed via one-way ANOVA followed by Dunnett’s multiple comparisons test. * *p* < 0.05.

Genes coding for neuropeptides that are involved in the central regulation of food intake in the rat hypothalamus, *Ghrh*, *Sst*, *Npy*, and *Agrp* [26], did not differ in expression in all experimental groups, except for *Npy* and *Agrp*. A significant increase in *Npy* expression (about a 4-fold increase) and for *Agrp* (about 3.5 times increase), was observed at the peak of the disease compared with the control rats (Figure 2).

### 2.3. EAE Affects Expression of Gh, Ghrhr, and Ghr in Pituitary

**Figure 3:**
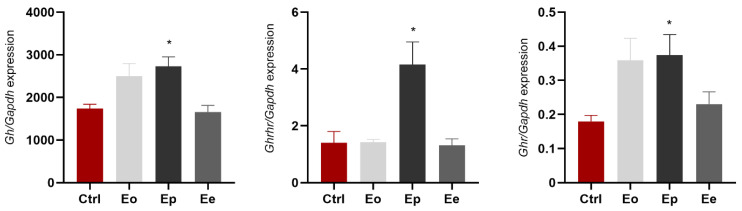
EAE upregulates relative gene expression of *Gh*, *Ghrhr* and *Ghr* in the pituitary gland at the peak of the disease. Levels of target genes are expressed relative to the expression of the *Gapdh* gene. Expression profiles were analyzed via RT-qPCR from RNA isolated from animals at Eo (light gray bars), Ep (dark gray bars), Ee (gray bars), and control animal (Ctrl, red bars). The data shown are mean ± SEM (n ≥ 5 animals/group), derived from a representative experiment. There are significant increases in the expression of these genes at the peak of EAE. The results were analyzed via one-way ANOVA followed by Dunnett’s multiple comparisons test. * *p* < 0.05.

We investigated whether EAE affects *Gh*, *Ghrhr*, and *Ghr* expression in the pituitary gland. *Gh* expression was increased at the onset of EAE (~40% compared to control, *p* = 0.06) and significantly upregulated at the peak of the disease (~60% compared to control, *p* = 0.009). At the end of the disease, *Gh* expression was almost equal to that of the control group (Figure 3). *Ghrhr* expression showed a significant increase only at the peak of the disease (~3× compared to control, *p* = 0.002). *Ghr* expression had a tendency to increase at the onset (~2× compared to control, *p* = 0.0687) and significantly increased at peak disease (2× compared to control, *p* = 0.0271; Figure 3).

### 2.4. EAE Does Not Alter Stereological Parameters in Somatotrophs during EAE

**Figure 4:**
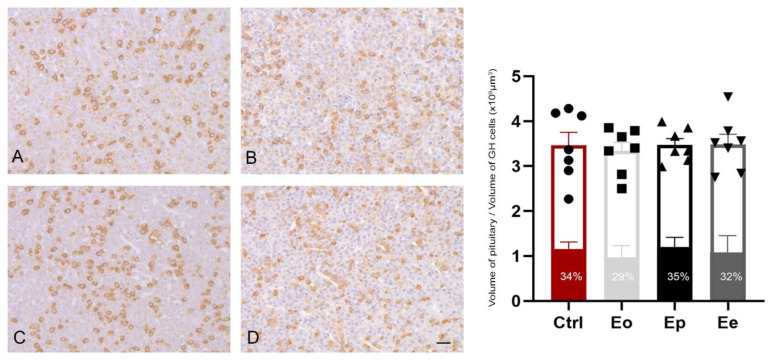
Immunohistochemistry of GH cells in pituitary sections from EAE rats with supporting quantitative data during the course of EAE. Representative micrographs display GH immunoreactivity in the pituitary gland in healthy (**A**) control rats (Ctrl, red bar) and animals sacrificed at the (**B**) onset (Eo, light gray bar), (**C**) peak (Ep, black bar), and (**D**) end (Ee, dark gray bar) of the disease. Nuclei are counterstained with hematoxylin. The scale bar applicable to all micrographs is 20 µm. Morphometric parameters were analyzed using stereological analysis of GH cells. The ratio of the pituitary volume (white bars) and volume of GH cells (filled bars) were measured from at least six sections, from seven different animals per EAE phase. Results are given as means ± SEM; analyzed using Kruskal-Wallis followed by Dunn’s multiple comparisons test.

In all experimental groups of rats, GH cells in the pituitary gland were intensely and uniformly stained. The immunopositive GH cells were oval or pyramidal, arranged singly or in groups (Figure 4A–D). Morphometric measurements of the pituitary gland during all EAE phases revealed no significant differences in the volume of the pituitary glands, volume of immunopositive GH cells, and the percentage of GH cell volume to total pituitary volume.

### 2.5. Increased GH and Decreased IGF-1 Circulatory Levels Associate with Neuroinflammation

Serum GH levels were significantly increased by more than 3.5-fold only at the peak of the disease (*p* = 0.0041). At the same time, IGF-1 levels began to decrease at the onset of the disease (*p* = 0.0262), with this level significantly decreasing at the peak of the disease (~30%, *p* < 0.0001) (Table 2).

### 2.6. EAE Alters Ghr and Igf1r Expression in Liver

**Figure 5:**
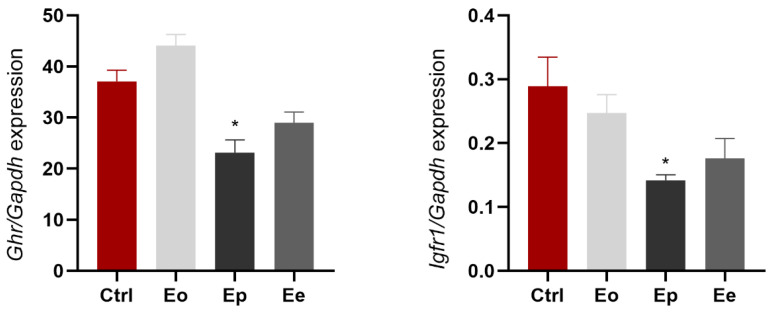
EAE affects *Ghr* and *Igf1r* gene expression in the liver. Levels of target genes are expressed relative to the expression of the *Gapdh* gene. Expression profiles were analyzed via RT-qPCR from RNA isolated from animals at Eo (light gray bars), Ep (dark gray bars), Ee (gray bars), and control animals (Ctrl, red bars). The data shown are mean ± SEM (n ≥ 5 animals/group), derived from a representative experiment. *Ghr* and *Igf1r* were down-regulated at the peak of EAE. The results were analyzed via one-way ANOVA followed by Dunnett’s multiple comparisons test. * *p* < 0.05.

The liver plays a crucial role in GH signaling and IGF1 production. Normal or elevated GH levels can lead to reduced responsiveness to GH signaling in the liver. Therefore, we investigated the expression of *Ghr* and *Igf1r* in the livers of control and immunized rats during EAE. We found that *Ghr* expression decreased significantly at the peak of the disease (37%, *p* = 0.0009) and at Ee (~22%, *p* = 0.0482) (Figure 5). *Igf1r* expression was significantly decreased at the peak of the disease (~2× compared to control, *p* = 0.0089) and had a tendency to return to the control levels at the end of the disease (Figure 5).

## 3. Discussion

The aim of this study is to investigate the effects of EAE on the GH/IGF-1 axis. We used the established animal model of multiple sclerosis to screen out a profile of the GH/IGF-1 axis in female EAE rats. To our knowledge, the present study is the first to show a multifaceted interplay between gene and protein expression at different levels of the GH/IGF-1 axis during EAE in female rats.

In this study, we found increased GH and decreased IGF-1 circulating levels associated with downregulated *Ghr* and *Igf1r* expression in the liver during the most neurologically severe phase of EAE, when body weight loss is extreme, corresponding to a state consistent with GH resistance. The results show that the gene expression of *Ghrh* and *Sst* in the hypothalamus did not change, while the expression of *Npy* and *Agrp* increased. At the pituitary level, the *Gh*, *Ghrhr* and *Ghr* genes were upregulated, while the size of somatotroph cells remained unchanged.

Growth hormone resistance is a condition in which the body’s cells do not respond appropriately to the action of growth hormone; it is a complex condition with several underlying causes, like malnutrition and inflammation [27,28]. Previously, it has been shown that in chronic inflammatory conditions, like sepsis, increased GH and decreased IGF-1 levels are linked with disease severity, suggesting that these conditions may be termed GH resistance [29,30]. Indeed, Zhao et al. demonstrate that proinflammatory cytokines, such as TNF-α, IL-β and IL-6, have a role in mediating chronic inflammation-induced GH resistance, where these cytokines lead to the inhibition of GHR expression in the liver [31].

In addition, it has been suggested that the state of GH resistance is an adaptive response to reduced energy intake due to poor nutrition [27]. Previously, nutritional status has been shown to dictate the effects of GH [32], with normal or elevated GH levels during prolonged nutrient deprivation and concomitant decreases in serum IGF-1 levels [32,33,34]. The observed reduction of IGF-1 level in serum is affected by a decreased *Igf1r* mRNA level in the liver, where most circulating IGF-1 is produced [35,36]. In addition, it was shown that hepatic *Ghr* mRNA levels are also down-regulated due to low IGF-1 serum levels and decreased GH binding to the membrane [36,37]. These results are consistent with those obtained in this study. In our study, the severity of EAE is positively correlated with the greatest decrease in body weight, as previously observed in several of our studies [6,38]. Furthermore, we demonstrated reduced expression of *Ghr* and *Igf1r* in the liver at the peak of the disease when body weight loss is most severe, resembling the state of GH resistance. EAE-associated weight loss occurs at the onset and peak of the disease when animals reduce or refuse food intake.

The hypothalamus plays a crucial role in regulating energy balance and feeding behavior, and inflammation within this brain region can disrupt these processes, previously shown in EAE [38,39]. Genes coding for neuropeptides that are involved in the central regulation of food intake in the rat hypothalamus, *Ghrh* and *Sst* [26], did not differ in expression in all experimental groups. Previously, it was shown that the pattern of GH secretion does not always correlate with altered gene expression of *Ghrh* and *Sst* in adult rodents [40]. However, the expression of *Npy* and *Agrp* increase at the peak of disease, quite the opposite to *Pomc* expression which we have previously shown to be reduced at the same time point [6]. NPY and AgPR are primarily known for their orexigenic effects, as they are potent and long-lasting appetite stimulators. They act to promote food intake and energy storage, particularly during times of negative energy balance or stress [41]. It has been shown that NPY is involved in the control of both food intake and GH secretion [42]. NPY and AgRP-expressing neurons become active during fasting, as part of the body’s response to promote food-seeking behavior and increase energy intake [43], thus the observed increase could be caused by the weight loss of the animals at the peak of the disease. This drives animals to seek food, so when food is consumed, like at the end of disease, *Npy* and *Agrp* levels typically decrease as part of the body’s response to food intake. This decrease in *Npy* and *Agrp* levels is probably associated with feelings of satiety and reduced hunger.

Interestingly, we found an increased gene expression of *Gh*, *Ghr*, and *Ghrhr* in the pituitary gland during the peak of the disease, while the volume of GH cells remained unchanged during all EAE phases, implying that functional changes occur in the pituitary gland during the disease without significantly altering the morphometry of the cells.

Previously, it has been reported that there is a negative correlation between levels of IGF-1 and ghrelin, with low IGF-1 levels inducing the synthesis and secretion of ghrelin [44]. We did not measure ghrelin levels, but in this case we hypothesize that it is elevated at the peak of EAE, as ghrelin levels also increase after food deprivation and after many forms of weight loss [45], as shown here in EAE. It has been proposed that ghrelin decreases plasma levels of IGF-1 and thus inhibits the negative effect of IGF-1 on GH secretion and acts directly on somatotrophs [44,46], which seems plausible in our experimental model. In addition, acute inflammation with subsequent weight loss leads to a negative energy balance, whereby ghrelin can influence the orexigenic centers in the hypothalamus to conserve energy. At the same time, the GH level rises, which is beneficial for the body, as GH is responsible for energy production by activating the breakdown processes in adipose tissue and the liver. At the end of the disease, after the clinical symptoms and inflammation have subsided, there is an increase in body weight and a normalization of GH and IGF-1 levels in the serum. In general, increased GH levels at the peak of disease fail to stimulate IGF-1 production effectively, and this state did not result from altered hypothalamic control (*Ghrh* and *Sst*), but rather pituitary control followed by the reduced IGF negative feedback inhibition. The increased GH levels in the serum influence a decrease in expression and secretion of IGF-1, which could explain the lack of the feedback inhibitory effect on pituitary GH secretion in the fasting state. We would like to point out that this study was conducted in female rats, as marked physiological, endocrine, and metabolic sex differences have been found in autoimmune diseases, including EAE [38], suggesting that the GH response to EAE may be different in male rats.

Overall, our results suggest that EAE associated with significant body weight loss induces changes in the blood circulation that affect the liver. Under conditions where resources are limited or the body is under stress, the body may prioritize essential physiological functions over growth-related processes. We hypothesize that our rats develop a state resembling GH resistance during the acute phase of EAE, probably as an adaptive response to ongoing disease. The rats may direct their resources towards critical functions rather than growth. This could be related to changes in the utilization of energy sources during the disease. It should be noted that the model presented in this study is self-resolving, i.e., after the clinical symptoms are reversed, most functions return to control levels. With regard to possible future therapeutic strategies, we therefore suggest that a possible involvement of the GH/IGF-1 axis should be investigated in detail.

## 4. Material and Methods

### 4.1. Animals

Breeding and husbandry of female Dark Agouti (DA) (RRID:RGD_21409748) rats was performed at the local animal facility. All experimental work was performed in accordance with the relevant national legislation on the use of animals for research of the Directive of the Council of the European Communities (2010/63/EU) and complied with the Law on Animal Welfare of the Republic of Serbia (Official Gazette of the Republic of Serbia, No. 41/2009). The experiments were approved by the local Ethics Committee of the Institute for Biological Research “Sinisa Stankovic,” (approval No 01-09/19) and Ministry of Agriculture, Forestry and Water Management of the Republic of Serbia, Department for Animal Welfare (approval, 323-07-05970/2020-05). The directive ARRIVE for better implementation of the “3Rs” was implemented to reduce the distress in animals during EAE experiments. Animals were kept under regular laboratory conditions with a 12 h dark–light cycle, constant temperature and humidity, and free access to laboratory food and water.

### 4.2. EAE Induction

To induce EAE, rats were subjected to brief CO_2_ anesthesia to minimize pain during inoculation of syngeneic rat spinal cord homogenate (SCH) emulsified with complete Freund’s adjuvant (CFA-F5881; Sigma, St. Louis, MO, USA), administered subcutaneously (100 µL) into both hind feet. Animals were observed every day during the experiment and evaluated for neurological deficits. Clinical severity was graded according to a predetermined scale using the following criteria: 0, asymptomatic; 1, tail atony—complete loss of tail tone; 2, hindlimb paraplegia; 3, complete hindlimb paralysis; 4, hindlimb paralysis/moribund; and 5, dead. At a score of 3, rats were given food and water on the floor of each cage to facilitate access, whereas the human endpoint was set at a score of 4 for two consecutive days. In addition, the rats were measured daily for body weight. Several parameters for the severity of the disease were assessed: duration of disease (the mean number of days the rats had a clinical score >1); duration of paralysis (the mean number of days the rats had a score ≥3); maximum severity score (the mean of the maximum clinical score that each rat in a group developed over the course of the experiment); and cumulative disease index (sum of daily clinical scores ≥3 over the time for the rat observed between day 10 and day 29).

### 4.3. Tissue Preparation

Rats were euthanized via gradual asphyxia in a CO_2_ chamber at three time points representing three phases of disease progression: onset (Eo), peak (Ep), and end (Ee) of EAE, along with control rats (Ctrl) in the diestrus phase of the estrous cycle. For gene expression studies, the hypothalamus and pituitary gland (n ≥ 6/group) were manually dissected at the respective time points after perfusion with 50 mL of ice-cold phosphate buffer and immersed in RNAlater^®^ RNA Stabilization Solution (Ambion™, Applied Biosystems by Thermo Fisher Scientific, Waltham, MA, USA), and stored at −80 °C until further processing.

For immunohistochemical studies, all groups were transcardially perfused with 50 mL ice-cold phosphate buffer, and the pituitary glands were placed in Bouin’s fixative for 48 h. Tissue was then immersed in ascending ethanol solution (30–100%), submerged in xylene and embedded in paraffin. Serial sections of the pituitary glands (5 µm thick; n = 7/group) were obtained with a rotary microtome (RM 2125RT, Leica Microsystems, Gmb, Solms, Germany).

Blood obtained via cardiac puncture was centrifuged at 5000× *g* for 15 min to obtain serum, and all samples were stored at −80 °C. Serum was collected from at least six animals per group in the control group (n = 6) and EAE animals (n = 5 or 6/group), and ELISA assays were performed.

### 4.4. Immunohistochemistry

To perform immunohistochemistry, sections were deparaffinized and rehydrated in water. After washing in PBS, sections were incubated in 0.3% hydrogen peroxide in methanol for 30 min to saturate endogenous peroxidase activities. The slides were incubated with 10% normal donkey serum for 30 min to block nonspecific binding sites. Then, the sections were treated overnight (at 4 °C) with a rabbit antigrowth hormone antibody (obtained from Dr. A. Parlow, National Institute of Diabetes and Digestive and Kidney Diseases, National Hormone and Peptide Program, Torrance, CA, USA) at a dilution of 1:1000. After washing with PBS, the sections were incubated with a donkey antirabbit-HRP antibody (1:200, Santa Cruz Biotechnology, Santa Cruz, CA, USA) for 2 h. The antigenic sites were then detected via incubation with 3,3′-diaminobenzidine (DAKO, Glostrup, Denmark). Sections were then dehydrated, cleaned, and mounted with DPX embedding medium (Fluka, Buchs, Switzerland). To ensure specificity of staining, negative controls (without primary antibodies) were performed in parallel.

All stereological analyses were performed using a workstation comprising a microscope (Olympus, BX-5; Olympus Microscopy, Tokyo, Japan) with a microcator (Heidenhain MT1201, Heidenhain, Chino, CA, USA) to control movements in the z-direction (0.2 μm accuracy); a motorized stage (Prior) for stepwise translation in the x-y direction (1 μm accuracy); and a CCD video camera connected to a 19″ PC monitor. The entire system was controlled with the new CAST stereology software package (VIS—Visiopharm Integrator System, ver. 2020.01.3.7887; Visiopharm; Denmark; www.visiopharm.com). The stereologic analysis included the absolute volume of the pituitary gland and the volume occupied by the immunopositive GH cells. Volumes were estimated according to the Cavalieri principle [47]. Sampling of pituitary sections was systematically uniform from the beginning (every 40th section from each tissue block was analyzed). The mean section thickness was estimated using the block advance (BA) method [48]. We found no deviation from the microtome set size of 5.0 μm. The total volume (mm^3^) of the pituitary gland and the volume of the immunopositive GH cells were determined by multiplying the sum of the areas by the distance between the sections (200 μm) according to the following formula:

$$\bar{V}=a(p)\cdot BA\cdot \sum_{i=1}^{n}Pi$$

where a(p) is the area assigned to each sampling point, BA (block advance) is the mean distance between two consecutively examined sections (real section thickness 5 μm × 40), and ΣPi is the sum of points hitting a given target. The microscopic images shown were acquired using a Zeiss Axiovert microscope (Carl Zeiss GmbH, Vienna, Austria).

### 4.5. ELISA

GH levels were obtained using a Rodent GH ELISA kit (Endocrine Technologies Inc., Newark, CA, USA) and the amount of IGF-1 was determined in serum via an immunoassay Mouse/Rat IGF-1/IGF-1 Quantikine ELISA Kit (cat. no. MG100, R&D Systems, Minneapolis, MN, USA) according to the manufacturer’s instructions.

The sensitivity of the GH kit is 0.2 ng/mL and the IGF-1 kit is 8.4 pg/mL. The absorbance was read at 450 nm with wavelength correction set to 570 nm using an automated ELISA plate reader (Titerteck Multiscan, ICN Pharmaceutical, Costa Mesa, CA, USA). To determine the GH level, calculation was performed using a four-parameter logistic curve fitting program and for the IGF-1 level, calculation was performed using a standard curve by plotting the mean absorbance for each standard on the *y*-axis against the concentration on the *x*-axis and drawing a best fit curve through the points on the graph.

### 4.6. Quantitative Real-Time PCR

Total RNA was extracted from the hypothalamus and pituitary gland of all experimental animal groups. Isolation was performed using the RNeasy Mini Kit (QIAGEN, Hilden, Germany). RNA (1 µg) was reverse transcribed using a High Capacity cDNA Reverse Transcription Kit (Applied Biosystems by Thermo Fisher Scientific, Waltham, MA, USA). Quantitative real-time PCR (RT-qPCR) analysis was performed with Power SYBR^TM^ Green PCR Master Mix (Applied Biosystems by Thermo Fisher Scientific) and primers designed on specific targets using a QuantStudio™ 3 Real-Time PCR System (Applied Biosystems by Thermo Fisher Scientific). Relative expression of mRNA (Table 3) was normalized to *Gapdh* as the reference gene and calculated based on the ∆∆Ct method. Results are expressed as mean ± SEM and were performed in 6 replicates per group from 2 independent experiments.

### 4.7. Statistical Analysis

All statistical analyses were performed using GraphPad software (RRID:SCR_002798, GraphPad Prism version 8 for Windows, GraphPad Software, La Jolla, CA, USA). The significance of the difference in means between groups was calculated using the one-way ANOVA test followed by the Dunnett post hoc test where the groups had passed the normality test, or the Kruskal–Wallis test followed by the Dunn’s post hoc test. Quantitative data were expressed as mean ± SEM, and values of *p* ≥ 0.05 were considered statistically significant.

## Figures and Tables

**Table 1:** Parameters of clinical severity of EAE.

Duration of Disease (Days)	Duration of Paralysis (Days)	Maximal Clinical Score	Cumulative Disease Index
8.3 ± 1.3	2.2 ± 0.4	2.9 ± 0.1	11.7 ± 1.5

**Table 2:** Serum GH and IGF-1 levels during EAE.

	Ctrl	Eo	Ep	Ee
GH (ng/mL)	4.2 ± 3.53	4.8 ± 4.03	15 ± 6.56 *	6.7 ± 2.52
IGF-1 (ng/mL)	1357 ± 346.30	842.67 ± 111.60	731.11 ± 95.34 *	2418.2 ± 337.46 *

* *p* < 0.05.

**Table 3:** Primer list.

Gene Symbol	Forward Primer Sequence	Reverse Primer Sequence	Accession Number
*Gapdh*	CAACTCCCTCAAGATTGTCAGCAA	GGCATGGACTGTGGTCATGA	NM_017008.4
*Gh*	GCACAAGGCAGAGACCTACC	CAAAGTGTAGGGGTGGCAGT	NM_001034848.2
*Ghr*	CCAACTCCCCTCTACACCAA	GGGCTAACCCTGCCTTAATC	NM_017094.1
*Ghrh*	GGGTGTTCTTTGTGCTCCTC	GCAGTTTGCGGGCATATAAT	NM_031577.1
*Ghrhr*	CTCTGCTTGCTGAACCTGTG	GACGAGTTGTTGGTCCCCTC	NM_012850.2
*Igf1r*	TCCCAAGCTGTGTGTCTCTG	CTCCGTTGTTCCTGGTGTTT	NM_052807.2
*Sst*	GATAGCGGCTGAAGGAGACG	CAAAGCCAGGACGATGCAGA	NM_012659.2
*Npy*	TACTACTCCGCTCTGCGACA	GGGCATTTTCTGTGCTTTCT	NM_012614.2
*Agrp*	TGTGTAAGGCTGCACGAGTC	AGTACCTAGCTTGCGGCAGT	NM_033650.1

## Data Availability

All reported data have been provided as part of the submitted article.
